# 2351 Environmental barriers and facilitators of health care access and utilization for elderly stroke survivors

**DOI:** 10.1017/cts.2018.254

**Published:** 2018-11-21

**Authors:** Allison Brenner, Lesli E. Skolarus, Philippa J. Clarke, James F. Burke

**Affiliations:** University of Michigan School of Medicine

## Abstract

OBJECTIVES/SPECIFIC AIMS: This study will use face-to-face interviewers with Medicare-eligible stroke survivors, and adult caregivers of stroke survivors, to extend the aims of a quantitative study on healthcare utilization in elderly stroke survivors. The objective of this research is to better understand, in more detail, relevant barriers and facilitators to accessing healthcare among older stroke survivors. The ultimate goal of this research is to develop strategies to improve access to healthcare, such as home modifications; changes to the neighborhood physical environment; or interventions at the provider/service level. This research will also serve as a precursor for future intervention work that will be proposed as a part of a K01 proposal. METHODS/STUDY POPULATION: Participants were recruited from Ann Arbor and Flint, MI using an existing academic-community partnership as well as through the University of Michigan Stroke Clinic. A total of 8–10 stroke survivors and 1–2 caregivers were recruited through the partnership and clinic records, as well as some use of snowball sampling to obtain a socially, economically, and racially representative sample. Participants must be 65+ years old, eligible for Medicare, living in the community, identify as either White or Black, and have no major cognitive/language deficits that jeopardize informed consent. Face-to-face interviews were conducted, and open-ended questions emphasized environmental barriers and facilitators to accessing healthcare, with a focus on social and physical barriers in the home and neighborhood. Interviews were audio recorded and transcribed, and field notes from 1 to 2 sources were also documented and will be used to triangulate the data and increase coding validity. Audio recordings will be reviewed multiple times and quotes relevant to the research questions and underlying theoretical framework will be transcribed verbatim. The transcripts will be analyzed using thematic coding based on literature and the study objectives and hypotheses. I will identify primary themes related to environmental barriers and facilitators to accessing healthcare among the stroke-survivors. RESULTS/ANTICIPATED RESULTS: Preliminary results suggest that participants are primarily concerned about the social environment. Several interviews revealed that stroke survivors felt socially isolated and were often hesitant to ask for help because they did not want to be a burden on their family and friends. Transportation to appointments was also identified as a barrier due to the fact that many people are no longer able to drive, yet are not comfortable navigating other forms of transportation. We expect to identify additional physical and social environmental challenges to both health care utilization and well-being more generally, among older stroke survivors. Anticipated themes may include: barriers in the physical environment such as transportation to care and services, social support and social environmental factors to support feeling safe leaving home to access care. DISCUSSION/SIGNIFICANCE OF IMPACT: Despite the physical and economic burden of stroke, and attempts to improve outcomes for stroke survivors living in the community, stroke survivors have high rates of disability and unmet medical and psychological needs. The results from this research are anticipated to directly inform future partnerships and intervention in these, or in similar communities. Understanding how the environment influences access to healthcare for elderly stroke survivors is essential if we want to increase recommended preventative care and treatment in this vulnerable population with unique healthcare needs. The results of this study will be used to directly inform the aims and methods for other translational research projects, including a K01 proposal, in which I will develop and pilot a community-based intervention to ameliorate environmental barriers and enhance facilitators of access to healthcare for older, disabled adults.

